# Varicella zoster virus immediate early protein 61 blocks the IFN-β pathway by degradation the activited IRF3

**Published:** 2011-01-10

**Authors:** Zhu Huifang, Chunfu Zheng

**Affiliations:** 1 State Key Laboratory of Virology, Wuhan Institute of Virology, Chinese Academy of Sciences, Wuhan 430071, PR China

## 

Varicella zoster virus (VZV) open reading frame 61 (ORF61) is one of the four transcription regulated proteins, which is homologous to herpes simplex virus 1 (HSV-1) ICP0 and can partially complement the function of ICP0 in ICP0 deletion mutant HSV-1. Since ICP0 can inhibit the innate immunity in many levels such as IRF3 and PML, here we investigate the role of ORF61 in helping VZV evading IFN-β signal pathway. As the role of IFN-β in VZV infection has little been reported previously, our results demonstrated that IFN-β can limit VZV replication in Mewo cells and VZV infection can suppress the secretion of IFN-β in 293T cells and HeLa cells stimulated by SeV or poly(I:C). In addition, we try to explore the molecule mechanism by VZV to evade host innate immunity system especially the IFN-β pathway. We found that ORF61 can inhibit the activity of IFN-β promoter in 293T cells by reporter assays in the presence of SeV or poly I:C. The activity of ISRE (interferon-stimulated response elements, ISRE) promoter was also inhibited but not that of NF-κB promoter by ORF61, suggesting that ORF61 may interfere with the activity of IRF3. Ultimately, our results demonstrated that ORF61 degraded phosphorylation IRF3 via its E3 ubiquitin ligase activity. In one word, VZV ORF61 could inhibit IFN-β pathway and may play a critical role in VZV pathogenesis.

